# The Nobel Prize of Physiology or Medicine, 1923: controversies on the discovery of the antidiabetic hormone

**DOI:** 10.1007/s00592-023-02098-9

**Published:** 2023-06-02

**Authors:** Alberto de Leiva-Hidalgo, Alejandra de Leiva-Pérez

**Affiliations:** 1 Universitat Autònoma de Barcelona, Barcelona, Spain; 2 Fundació DIABEM, Barcelona, Spain

**Keywords:** Nobel Prize, Priority rule, Acomatol, Pancreina, Insulin

## Abstract

**Aims:**

To analyze the main contributions to the discovery of the antidiabetic hormone in the period between 1889, the year in which Oskar Minkowski demonstrated that complete pancreatectomy in dogs caused diabetes, and the year 1923, the date in which the clinical use of insulin was consolidated. A main objective has been to review the controversies that followed the Nobel Prize and to outline the role of the priority rule in Science.

**Methods:**

We have considered the priority rule defined by Robert Merton in 1957, which takes into account the date of acceptance of the report of a discovery in an accredited scientific journal and/or the granting of a patent, complemented by the criteria set out by Ronald Vale and Anthony Hyman (2016) regarding the transfer of information to the scientific community and its validation by it. The awarding of the Nobel Prize in Physiology or Medicine in October 1923 has represented a frame of reference. The claims and disputes regarding the prioritization of the contributions of the main researchers in the organotherapy of diabetes have been analyzed through the study of their scientific production and the debate generated in academic institutions.

**Main results and conclusions:**

(1) According to the criteria of Merton, Vale and Hyman, the priority of the discovery of the antidiabetic hormone corresponds to the investigations developed in Europe by E. Gley (1900), GL Zülzer (1908) and NC Paulescu (1920). (2) The active principle of the pancreatic extracts developed by Zülzer (acomatol), Paulescu (pancreina) and Banting and Best (insulin) was the same. (3) JB Collip succeeded in isolating the active ingredient from the pancreatic extract in January 1922, eliminating impurities to the point of enabling its use in the clinic. (4) In 1972, the Nobel Foundation modified the purpose of the 1923 Physiology or Medicine award to Banting and Macleod by introducing a new wording: "the credit for having produced the pancreatic hormone in a practical available form" (instead of “for the discovery of insulin”).

**Supplementary Information:**

The online version contains supplementary material available at 10.1007/s00592-023-02098-9.

## Alfred Nobel's Testament (1895)

**Fig. 1:**
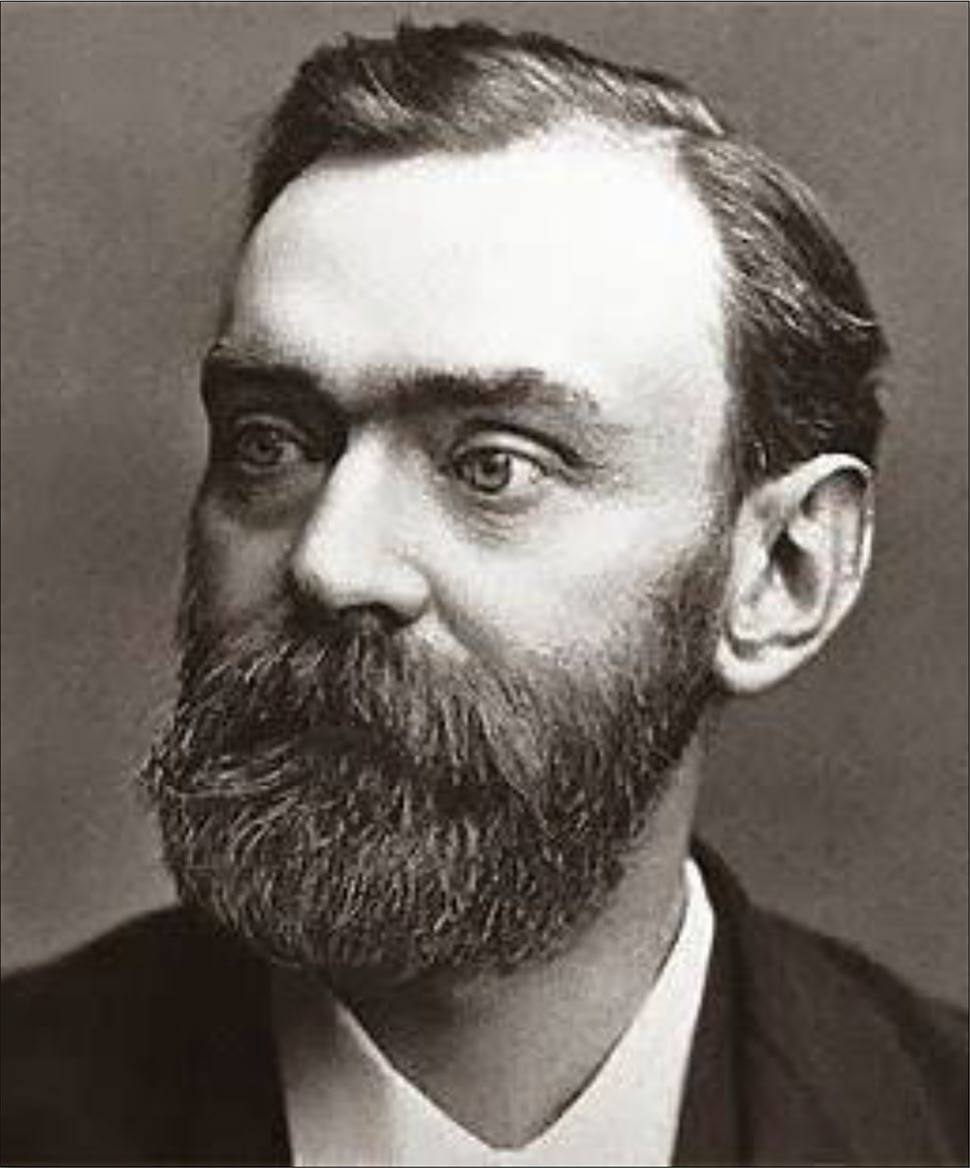
Portrait of Alfred Nobel. Unknown date and author [1]. Public domain

**Fig. 2:**
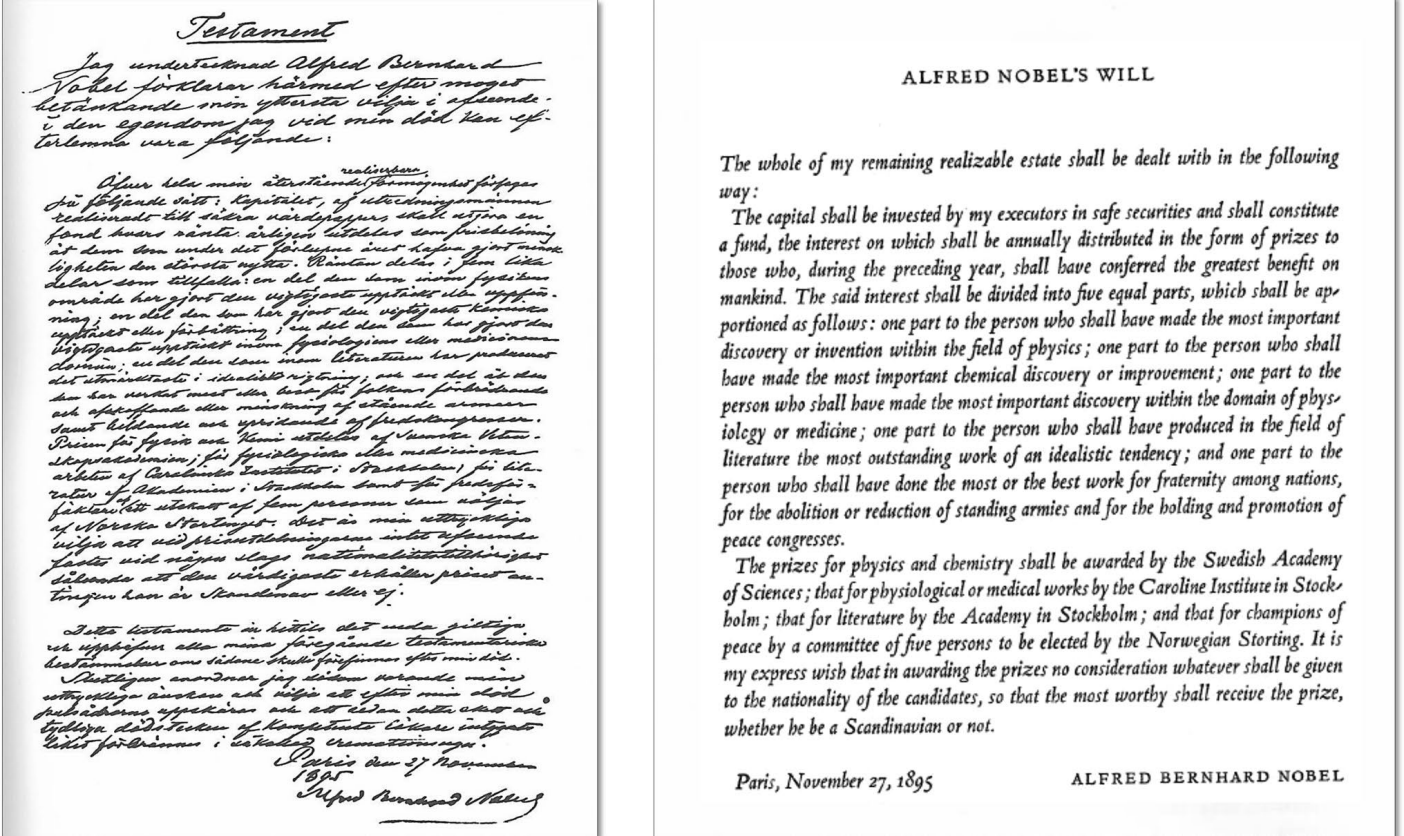
Testament of Alfred Nobel [2]. Public domain

On November 27, 1895, a year before his death from a cerebral hemorrhage, Alfred Nobel (1833–1896) dictated his will at the Swedish-Norwegian club in Paris, by which he bequeathed to his relatives (he did not marry or have children) a legacy of 100,000 crowns. The rest of his fortune (about 33 million crowns) was allocated to the annual award of the Physics, Chemistry, Physiology or Medicine, Literature and Peace Prizes (Figs. 1, 2) [1, 2].

## The award of the 1923 Nobel Prize in Physiology or Medicine

Jan Lindstein and Nils Ringertz have described in depth the procedure followed in the election of the Nobel Prizes in Physiology or Medicine in the period between 1901 and 2000 [3].

The activity regarding the awarding of the Nobel Prize in Physiology or Medicine is carried out by the Nobel Committee, elected by the Nobel Assembly from among its members, all of whom are professors at the Karolinska Institute. The Nobel Assembly is independent, both from the legal point of view and from the economic-administrative. Funding for the prizes comes exclusively from the Nobel Foundation. The Nobel Assembly has freedom to reward, indistinctly, areas in the broad field of biomedical research and clinical medicine. The prize is awarded for an extraordinary discovery and not for a scientific career of a lifetime. The concession must recognize a contribution or discovery of the highest level made during the previous year, a very difficult standard to meet, since the discovery must be published, known and evaluated by a group of internationally qualified researchers, a process that rarely lasts less than one year. To solve this dilemma, the pragmatic solution was to interpret that what matters is that *the benefit of the discovery has become evident to the members of the Nobel Assembly during the year preceding the date of the award.*

In the month of September of the year prior to the awarding of the prize, invitations are sent to some 3,000 scientists from non-Scandinavian universities and academic institutions chosen by a rotating system, to make proposals for appointments. Proposals must be made according to a model sent by the Nobel Committee. Scientists previously recipients of the Nobel Prize in Physiology or Medicine and professors at medical schools in the Nordic countries can exercise their right to make proposals each year. The deadline for submitting proposals is January 31 of the year of the award. Between the months of March and May, the Nobel Committee invites international experts to submit their reports. All candidates are evaluated by members of the Nobel Assembly and external reviewers; the evaluations are sent before the end of August. In September, the Nobel Committee sends its recommendations on potential candidates to the Nobel Assembly. In the month of October, the election takes place by majority vote. This decision is final, with no possibility of appeal. The laureates are immediately informed and the decision is announced at a press conference. The Nobel Prize award ceremony takes place on December 10 in Stockholm, during which the laureates receive a gold medal, a diploma and a monetary award.

### Influence of August Krogh in awarding the Nobel Prize to Banting and Macleod

Schack August Stenberg Krogh (1874–1949) received the Nobel Prize in Physiology or Medicine in 1920 "for his discovery of the regulatory mechanisms of capillary circulation".

**Fig. 3:**
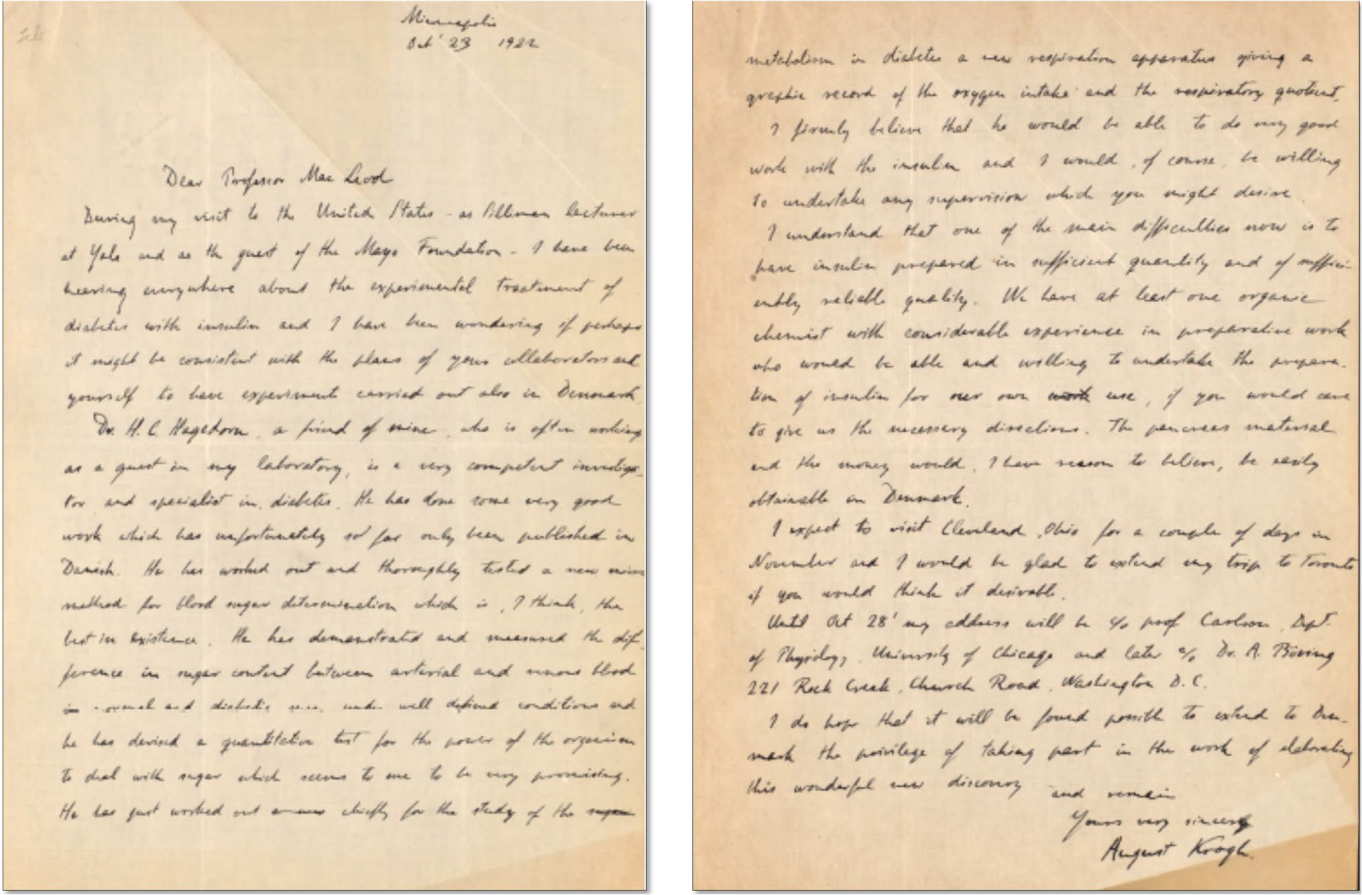
August Krogh sent this letter to JJR Macleod on October 23, 1922, proposing a possible collaboration with HC Hagedorn to extend insulin production and therapeutic trials to the University of Copenhagen (Archives of the University of Toronto Library)

His wife, Dr. Birte Marie Krogh, was diagnosed with diabetes mellitus in 1921, under the medical care of Dr. Hans Christian Hagedorn (1868–1971). In 1922, Krogh received an invitation to give several lectures in the USA. In November, in Boston, Dr. Elliot P. Joslin briefed him on research from the University of Toronto into the therapeutic benefits and purification process of insulin. On October 23, 1922, Krogh wrote to Macleod from Minneapolis, informing him of his interest in investigating the actions of the pancreatic extract and producing insulin in Denmark. Macleod immediately responded in favorable terms (Fig. 3).

Krogh decided to extend his trip, going to Toronto as a guest of Prof. JJR Macleod. He returned to Copenhagen in December 1922. Marie successfully started her insulin treatment in December 1922, and Krogh obtained the license from the University of Toronto to produce insulin in Scandinavia (Suppl. Fig. 1).

The day after their arrival in Denmark, Krogh and Hagedorn began an intensive investigative process. The first experiments were carried out in Hagedorn's own house and in the laboratory of the Institute of Zoology, directed by Krogh. Very soon, on December 21, 1922, they successfully obtained a small amount of insulin from bovine pancreas. Immediately, August Krogh and Hans Christian Hagedorn founded the Nordisk Insulin Laboratory company, with the financial contribution of August Kongsted (1870–1939), pharmacist and owner of the company Løvens Kemiske Fabrik (Leo Pharmaceutical Products). Kongsted financed the research and production process. In compensation for such an important contribution, the condition requested by Kongsted was to name the first manufactured product "Insulin Leo", which became available in the spring of 1923. The first Danish patient treated with “Insulin Leo” received his initial dose on March 13, 1923.

Prof. August Krogh and Prof. Göran Liljestrand (Executive Secretary of the Nobel Committee since 1918) were friends. Their relationship intensified after Liljestrand did an academic stay in Copenhagen. On January 20, 1923, Krogh wrote a letter to Göran Liljestrand, in the following terms (Suppl. Fig. 2):

As you understand from my discourse, it is my opinion that the discovery of insulin is of extraordinary both theoretical and practical importance and it will hardly surprise you that I intend to submit a nomination that the Nobel Prize be awarded to Dr. Banting and Professor Macleod.

The Nobel Committee for the award of Physiology or Medicine of the year 1923 consisted of 5 members, in addition to its president and executive secretary (Prof. Göran Liljestrand). The Archives of the Karolinska Institutet received within the stipulated period the joint proposal of August Krogh in favor of FG Banting and JJR Macleod, and the individual proposals of Francis G Benedict (Prof. of Physiology, Harvard University, Cambridge, Mass., USA) and Georg W Crile (Prof. of Surgery, University of Cleveland, Ohio, USA), on behalf of FG Banting; and Georg N. Stewart (Professor of Experimental Medicine, Case Western Reserve University, Cleveland) on behalf of JJR Macleod.

August Krogh reasoned his joint proposal of Macleod and Banting in the following terms:

With the information which I personally have obtained in Toronto, and which also, although less clearly so, emerges from the published works, one may conclude that the credit for the idea behind the work which led to the discovery undoubtedly goes to Banting, who is a young and apparently very talented man. However, he would definitely not have been able to carry out the investigations, which from the start and during all stages have been supervised by Professor Macleod.

Macleod's special contributions to experimental work had only been partially published before the January 31, 1923, deadline. Macleod, working alone, had located insulin in the pancreas of teleost fish, demonstrating its hormonal function; he had also conducted investigations of the insulin actions on intermediary metabolism and respiratory quotient. Written reports on the proposals were prepared by two members of the Nobel Committee, John Sjöqvist, Professor of Chemistry and Pharmacy, and Hans Christian Jacobeus, Professor of Internal Medicine. Sjöqvist focused primarily on its physiological importance. He discussed the work of several predecessors, especially Georg Ludwig Zülzer. In his conclusion, he supported the joint request of A. Krogh, accepting the suggestion of dividing the prize between Banting and Macleod. Banting had the original idea and the first initiative; Macleod was the leader of the scientific work. Jacobeus’ decision was more difficult because “Macleod’s contribution was not apparent from the consulted bibliography” [2]. He then continued: “Banting came to Macleod with his idea… it is very likely that the discovery would not have been made if Macleod had not supervised him, at least not as rapidly as is now the case… I am most prone to give Banting and Macleod a joint Nobel Prize” [4].

After the Nobel Assembly had discussed the matter, the recommendation was sent back to the Committee for reconsideration on October 11, 1923. The objection responded to the need to verify the foundations of Krogh’s joint proposal, based on his visit to Toronto [5].

The Committee met again, reaffirmed his recommendation and made an extensive written statement to finally conclude that “… it is not possible to make a more thorough investigation of this discovery and the relative contributions by Banting and Macleod, nor is it necessary” [6].

The Committee considered valid the argument that although the discovery corresponded, initially, to Banting's idea, Macleod's guidance was decisive for its successful fulfillment. Finally, the nineteen professors of the Karolinska Institutet cast their secret ballot, ruling that “The Nobel Award of Physiology and Medicine was jointly granted on October 25, 1923, to Frederick Grant Banting and John James Rickard Macleod, for the discovery of insulin, one year before” [7].

It is logical to assume that Krogh's influence on Macleod's candidacy for the Nobel Prize was manifest, particularly as Krogh was previously laureated in 1920 and was informed by Macleod in Toronto on the scientific activities carried out in situ, benefiting from the license of the University of Toronto to produce insulin in Denmark [8].

**Fig. 4:**
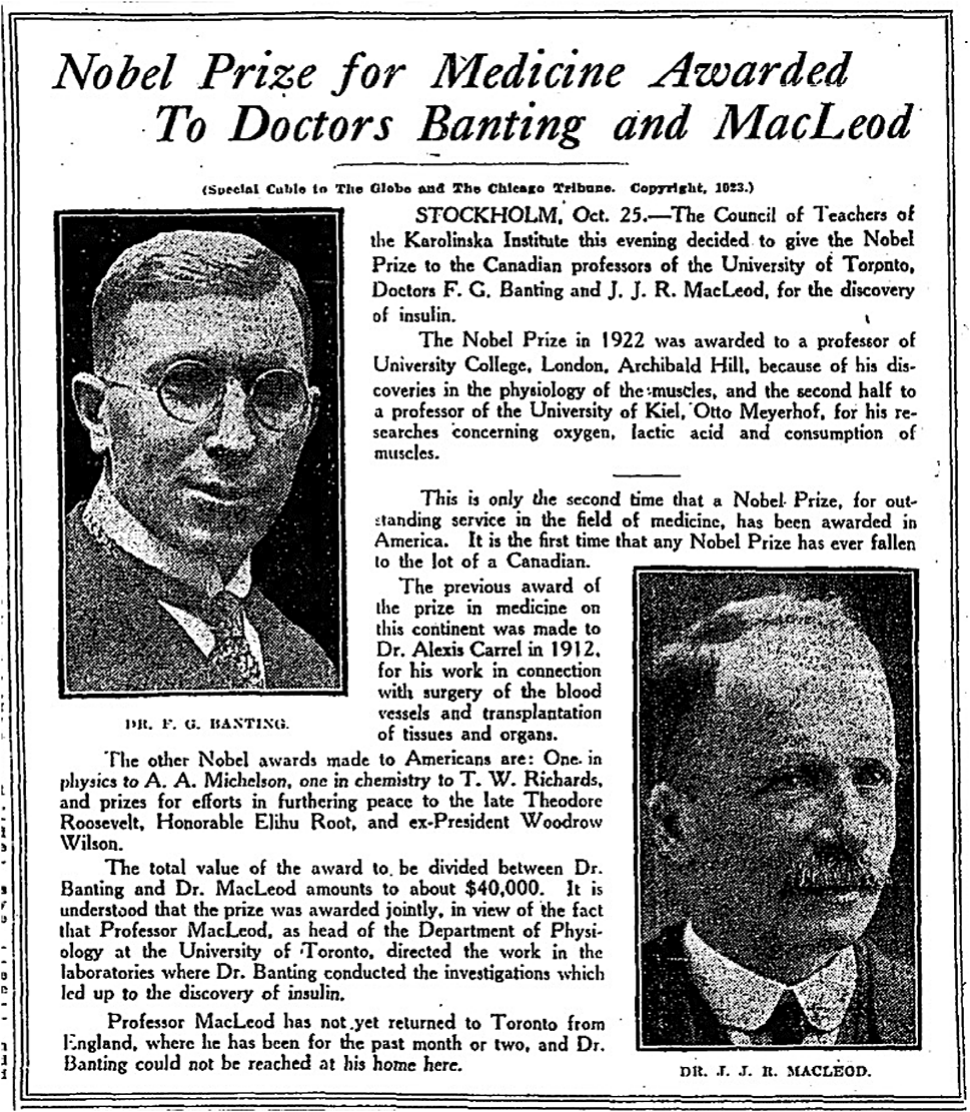
Front page of *The Globe* newspaper, October 23, 1923, reporting the award of the Nobel Prize in Medicine to Doctors Banting and Macleod

Frederick G. Banting learned of the Nobel Prize award on the morning of October 26, 1923, from the front page of The Globe newspaper (Fig. 4).

Banting was furious about having to share the award with JJR Macleod, and his first intention was to reject the prize. JG Fitzgerald, director of Connaught Laboratories, tried to persuade him. Banting’s answer was: “I will not accept the prize: I am going to cable Stockholm that not only would I not accept but that they and the old foggy Krogh could go to hell…I defied you to name one idea that had originated in Macleod’s brain – or to name one experiment he had done with his own hands”.

Fitzgerald was soon able to calm him and suggested he should meet Albert E. Gooderham, chairman of the Insulin Committee of the University of Toronto Board of Governors. Gooderham managed to convince Banting, arguing that other considerations deserved to be taken into account: “I must think of my country. What would the people of Canada think if the first Canadian to receive this honor were to turn it down? Banting did not say he will decide it immediately and better wait 24 h”. On October 26, Banting grabbed a notepad and wrote a telegram to Best (he was in Boston): “Nobel trustees have conferred prize on Macleod and me. You are with me in my share always” [9].

Banting decided to split 50% of the corresponding financial contribution with Charles H. Best. Macleod first heard the Nobel news on a steamship returning from Scotland to Canada. He decided also to split 50% of the corresponding amount with James B. Collip.

The monetary value of the prize in 1923 was 114,935 Swedish Crowns (SEK) (24,000 USD). This amount would be equivalent in 2021 to approximately 3,241,843 SEK (419,888 USD). In the years 2021–2022, the monetary value of the prize has reached 10 million SEK (1,103,148; USD; 964, 000 €) [10].

The University of Toronto celebrated the Nobel award with a special ceremony on November 26, 1923, in which both laureates, FG Banting and JJR Macleod, received the honorary degree of Doctor of Science. A banquet in the Great Hall of Start House that evening capped off the celebration with more than 400 guests representing the North American government, scientific, and medical professionals (Suppl. Fig. 3).

On December 10, 1923, Prof. J. Sjöquist, member of the Nobel Committee for Physiology or Medicine, delivered the Presentation Lecture for the Nobel Prize to Dr. Frederick Grant Banting and Professor John James Rickard Macleod “by the discovery of insulin”. In the absence of the laureates, the British Minister received the prizes from the hands of His Majesty the King, for future transfer to them [11].

Banting took all possible actions to make unpleasant Macleod’s life. The Scottish Professor felt he had to start legal actions against Banting, but he finally decided to leave the University and the city of Toronto in 1928. When the University held a farewell dinner for Macleod, Banting not only refused to attend, he requested that there be an empty place set for him at the table [5].

## Discovery of the pancreatic origin of diabetes: Oskar Minkowski

In April 1889, Oskar Minkowski, of the Department of Medicine at the University of Strasbourg (directed by Professor Naunyn), met Joseph von Mering in the library of the Hoppe-Seyler's Institute, University of Strasbourg. In 1886, von Mering had generated experimental diabetes with the administration of phloridzin and was then investigating the enzymatic function of the pancreas in the digestion of fatty foods.

Minkowski suggested that to achieve this goal it would be highly desirable to investigate the effects of total pancreatectomy and was able to overcome initial resistance from von Mering, who felt that such surgery was impossible to perform successfully. That same day they agreed that Minkowski would perform, with Mering's help, a complete pancreatectomy in the experimental laboratory of the Department of Medicine on one of the dogs at Hoppe Seyler's laboratory. After the first pancreatectomy, Mering did not participate in further research on this matter, directing his excellent academic career elsewhere. Minkowski reproduced the effects of complete pancreatectomy in three other dogs. The first two died, but the third survived and in less than 48 h presented glycosuria, polydipsia and polyuria. In the weeks following surgery, gradual weight loss was observed in spite of excessive energy intake, also showing persistent glycosuria, ketonuria and hyperglycemia.

Minkowski presented the first paper at the International Congress of Physiology in Basel. The first publication was a short communication in 1889 [12]. More extensive reports appeared in 1890, 1892 and 1893 [13–15].

Minkowski and Naunyn had previously shown that beta-hydroxybutyric acid is an agent responsible for diabetic acidosis and that the concentration of carbon dioxide in the blood markedly decreases in this acute metabolic emergency [16].

Rolf Luft (1914–2007), Professor of Endocrinology, Karolinska Hospital, Stockholm, in asking who discovered insulin declared that “Minkowski was the first to show that diabetes is the consequence of the absence of the pancreatic substance carried by the bloodstream, which motivated all the subsequent activities aimed at extracting the antidiabetic hormone. It has been the most important original discovery in the history of diabetes” [17].

## Protests over the awarding of the Nobel Prize in Physiology or Medicine in 1923

**Fig. 5:**
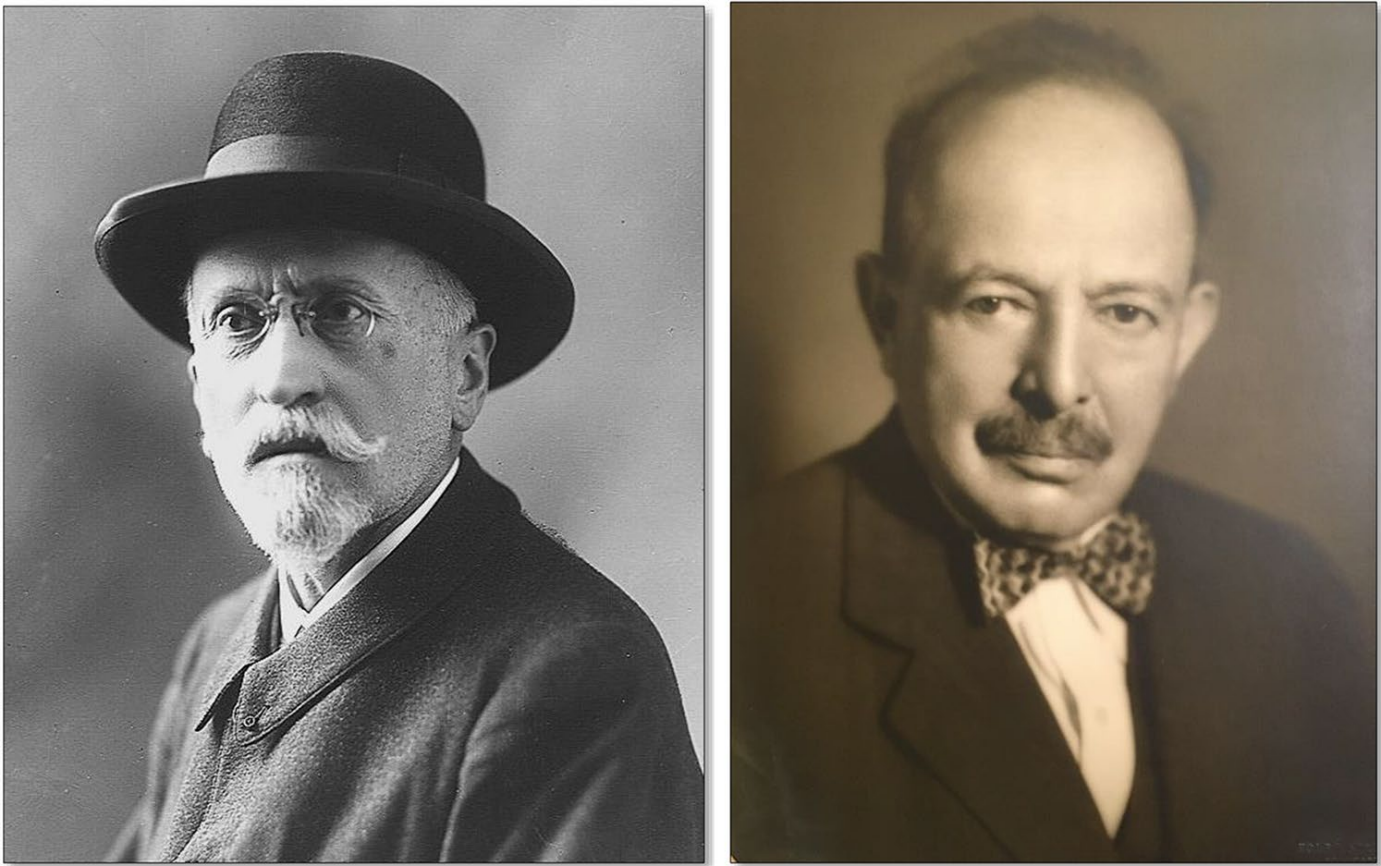
*Left*: Portrait of Marcel Eugène Émile Gley. Unknown author and date. Public domain. *Right*: Portrait of Georg Ludwig Zülzer. Courtesy of Viktor Jörgens (Jörgens V (2022). *Die Geschichte der Diabetesforschung—vom Opium zum Insulin*. Verlag Kirchheim Mainz)

**Fig. 6:**
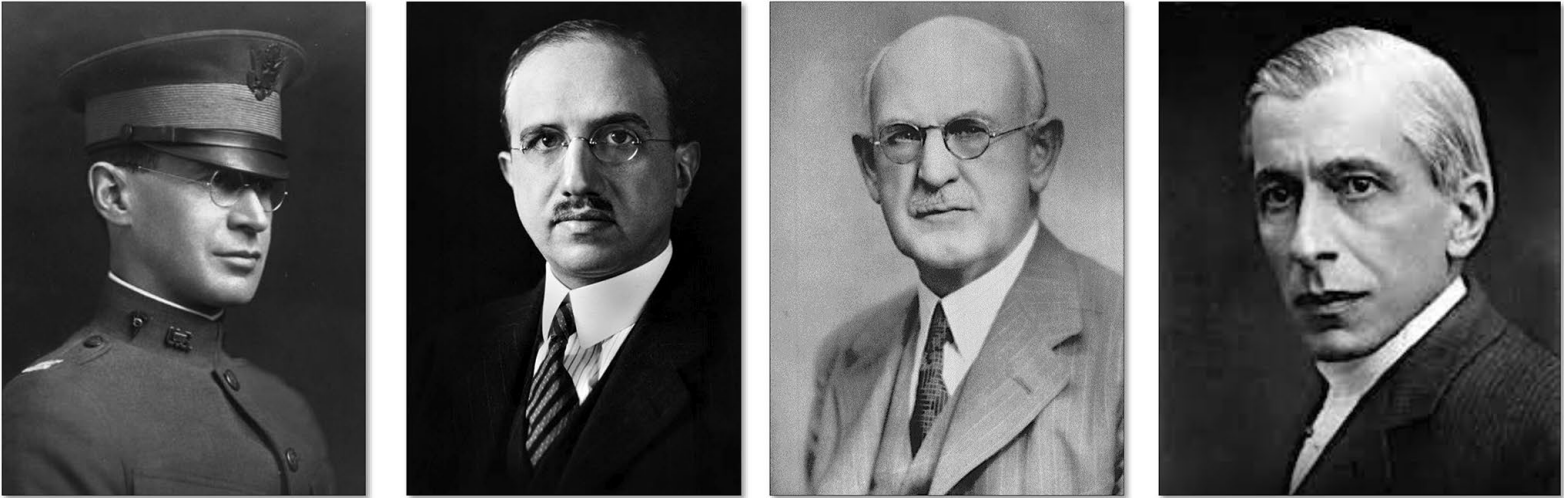
*From left to right*: Photograph of Ernest L Scott in 1917 in military uniform. Charles Best Papers (University of Toronto). Photograph of Israel S. Kleiner in 1915. Author: Louis Schmidt. *Source*: Rockefeller Archive Center. Portrait of John R. Murlin. Source: John Murlin Papers. Eskind Biomedical Library Special Collections, Vanderbilt University Medical Center. Portrait of Nicolae C. Paulescu. Unknown date and author. Academia Romana Filiala Cluj-Napoca. Public domain

The decision of the 19 signatories from the Karolinska Institutet was protested in subsequent weeks and months by at least four researchers, who claimed their priority in isolating the antidiabetic hormone: Georg Ludwig Zülzer (Berlin), Ernest Lyman Scott (Chicago), John Raymond Murlin (New York) and Nicolae Constantin Paulescu (Bucharest). Many years later, Charles Herbert Best would also claim the discovery (Figs. 5, 6) [18].

## Synoptic review of the main contributions toward the discovery of the antidiabetic hormone antedating the announcement of the development of insulin at the University of Toronto

### Oskar Minkowski (1858–1931)

A. Minkowski demonstrated that complete pancreatectomy was determinant of the generation of diabetes mellitus in the dog.
B. In addition, he developed liver and muscle analysis which demonstrated the disappearance of glycogen after experimental pancreatectomy (1889–1990) [13, 19].

### Emmanuel Charles Edouard Hédon (1863–1933)

A. Hédon characterized the dual role (exocrine-endocrine) of the pancreatic gland and introduced the original method of pancreatectomy in two stages (1891–1892) [20–22].

### Marcel Eugène Émile Gley (1857–1930)

A. Gley reported the presence of the *antidiabetic principle* in the extract of "sclerosed pancreas" (1891) (Fig. 5) [23]
B. Gley was the first to reveal that the parenteral administration of extracts of "sclerosed pancreas" reduces glycosuria and symptoms of experimental diabetes (1900) [24].

### Georg Ludwig Zülzer (1870–1949)

A. Georg Ludwig Zülzer demonstrated that the pancreatic extract prevented the diabetogenic effect of adrenalin in experimental animals (1907) and reduced the glycosuria of depancreatized dogs (Fig. 5) [25, 26].
B. Zülzer was the first to report the partial success achieved by the treatment with pancreatic extracts in a group of eight patients with diabetes. The treatment was associated with adverse side effects (1906–1908) [27, 28].
C. Zülzer obtained patents in Germany, Great Britain and USA for **acomatol,** his original formulation of pancreatic extract (1908–1912) [29].
D. Zülzer and Camille Reuter managed to minimize the toxicity of the pancreatic extract and increase its power to such an extent that it generated severe hypoglycemia (years 1913–1914) in the dog [30]. The start of the First World War and the decision of Hoffmann La Roche to cancel the financial support precluded the continuation of the research project [28, 29]. In 1923, Zülzer claimed the priority of the discovery of insulin in a document released by an international agency (Suppl. Fig. 4) [31] and in an article published in *Medizinische Klinik* (Suppl. Fig. 5) [32].

I feel entitled to claim priority in this discovery (…) because in the German scientific literature, partly due to ignorance, the role I played in the discovery was not always correctly perceived [26]

### Ernest Lyman Scott (1877–1966)

Ernest L. Scott (Fig. 6) defended in 1911 his M.Sc. thesis entitled “The effect of pancreas extract on depancreatized dogs, carried out in the Department of Physiology at the University of Chicago, a manuscript in which he described his original procedures for obtaining aqueous and alcoholic extracts of pancreas, which he administered intravenously to depancreatized dogs. The injection of the aqueous extract determined the reduction in glycosuria, associated with a decrease in the urinary D/N ratio in two out of the three investigated animals. For Scott, the active principle present in the pancreatic extract was of a protein nature [18, 33, 34].

In the summer of 1911, Scott finished the thesis manuscript and delivered three copies to his supervisor Anton J. Carlson, head of the Physiology department. Scott left Chicago with his family in September to accept an academic position at the University of Kansas. Professor AJ Carlson submitted EL Scott's thesis manuscript to the *American Journal of Physiology* under Scott's signature as sole author, without consulting him. Furthermore, Carlson substantially modified the content of the manuscript. In Scott's original document, the first conclusion was: “There is an internal secretion from the pancreas controlling the sugar metabolism”, and the text of the second conclusion: “By proper methods this secretion may be extracted and still retain its activity”. However, in the final paragraphs of the article published in *Am J Physiol*, Carlson disavows Scott, modifying the essential result of the thesis: “It does not follow that these effects are due to the internal secretion of the pancreas in the extract (…). The pancreas extract may decrease the output of sugar from the tissues by a toxic or depressor action, rather than by a specific regulatory action of the pancreas” [35].

Scott did not have access to the manuscript until its publication in 1912. On May 3, 1964, he signed a document denying authorship of the article submitted by Carlson [36].

Scott claimed priority for the isolation of the antidiabetic hormone in a letter to the editor of the *Journal of the American Medical Association*, published in October 1923, in which he defended the pioneering nature of his experimental work. In his opinion, the procedures for making and administering the adult bovine pancreatic extract described by Banting, Best and Collip in their application to the US Patent Office reproduced his own experience [37].

Dickinson W. Richards (Nobel Prize in Physiology or Medicine, 1956), Professor of Medicine at Bellevue Hospital, University of Columbia, New York, and former student of Scott's, published the thesis in its original form in 1966 [38]. After Scott’s death, his widow, Aleita H.Scott, accessed the archives of the University of Chicago to retrieve the original documents related to this conflict [39].

### Israel Simon Kleiner (1885–1960)

Israel Kleiner (Fig. 6) received his PhD in Biochemistry at the University of Yale in 1909. Between 1910 and 1919, he worked at the Rockefeller Institute.

A. In collaboration with SJ Seltzer, Kleiner published in 1915 that pancreatic extracts lowered blood glucose in normal and pancreatectomized dogs intravenously infused with glucose, which suggested that the internal secretion of the pancreas contributed to the rapid disappearance of glucose from the general circulation [40].
B. Kleiner published in 1919 that intravenous administration of a pancreatic emulsion achieved a significant reduction in blood glucose in a series of depancreatized dogs and toxic effects did not occur [41].

### John Raymond Murlin (1874–1960)

A—John Murlin, PhD (Univ. Pennsylvania, 1901), Assistant professor at Cornell University Medical College (1909–1917) (Fig. 6), reported in 1913, in collaboration with B. Kramer, that parenteral administration of the pancreatic extract did not change the respiratory quotient in healthy dogs, generating uncertainty about previous observations.

They treated several dogs, depancreatized using Hédon’s method, by injecting them intravenously with pancreatic extract. The urine collected in twenty-four hours periods showed an increase in the dextrose:nitrogen ratio on the following days, and the marked fall in blood glucose levels lasted between four to ten hours. In parallel, they repeated the experiments with a double extract of dog’s pancreas and duodenal mucosa, observing much greater effects. Surprisingly, they found similar results when either the pancreas or the duodenum was extracted with Ringer’s solution made alkaline with sodium carbonate. As identical findings were exhibited on the same dog, the authors concluded that the reduced glucose excretion was due to a change in the permeability of the kidney and not to a hormonal effect. Additional calorimetric experiments showed no effect of the organic extracts on the combustion of sugar [42].

B—In 1917, John Murlin achieved the position of Professor of Physiology at the University of Rochester. His research team demonstrated in 1922 the adverse influence of elevated pH upon the metabolic effects of the administration of pancreatic extracts to depancreatized animals, which provided an explanation of the conflicting results of its previous publication in 1913 [43].

C—In the same year, the team headed by John Murlin demonstrated that the biological actions of the pancreatic extract in pancreatectomized dogs were only manifested after parenteral administration, other routes of administration being ineffective. In the majority of cases, they observed a biphasic blood glucose response consisting of an initial rise in blood glucose, followed by the expected lowering of the sugar in blood and urine. The lowest level of blood sugar was reached in 4 h from the time of injection, whether the extract was given into vein, peritoneum, muscle or under the skin [44]. Murlin attributed the initial rise in blood glucose to a substance of pancreatic origin he named glucagon [45].

D—In cooperation with C. Sutter, Murlin reported the case of a diabetic patient with ketosis treated at the Rochester General Hospital in July 1922 with pancreatic extract administered through a gastrointestinal catheter and by oral and subcutaneous routes. Only in this latter case could glycosuria and ketonuria be decreased. On July 26, 1922, blood glucose level went down from 513 to 241 mg/dL [29; pp. 168, 46].

### Nicolae Constantin Paulescu (1869–1931)

A. Nicolae Constantin Paulescu (Fig. 6) published in 1920 an original procedure for the complete pancreas ablation, the protocol to elaborate a successful pancreatic extract [47], and the effect of pancreas ablation on glycogen stores [48]. All experiments had been carried out before 1916.
B. Between April and June of the year 1921, Paulescu published four short papers covering nine experiments, showing the convincing results achieved by central and peripheral intravenous injections of the pancreatic extract on carbohydrate, protein and fat metabolism in depancreatized dogs [49].
C. Paulescu illustrated the time sequence of these metabolic changes [50], the relation between the amount of the administered extract and the observed results [51], and the provocation of hypoglycemia by the extract on the normal dog [52].
D. On August 31, 1921, he published a comprehensive article, describing a summary of experiments conducted over 20 years of research, showing the technique to achieve a sterile extract, the methods to estimate the metabolic effects, the clinical manifestations of untreated and treated depancreatized dogs, the informed autopsies, the results of control experiments, and the side effects of the pancreatic preparation that he named **pancreina** [53].
E. On April 12, 1922, Paulescu registered the patent of pancreina in the Romanian Ministry of Industry [18, 54].

Nicolae C. Paulescu's protest was probably the most acid and bitter. On February 5, 1923, he wrote to Frederick Banting, enclosing his 1921 publications on the treatment of experimental diabetes. Banting never replied. On November 6, 1923, Paulescu sent a letter to the President of the Nobel Commission; in the letter he claimed against the award of the Nobel Prize to Banting and Macleod. According to Paulescu, the Toronto team had violated his intellectual property rights and his discovery of the antidiabetic hormone had been stolen by the Canadian researchers. For this reason, he demanded from the Commission a conduct consistent with the application of the principles of justice [18, 55].

Shortly before dying, Paulescu declared (1931) [18]:

Formerly I believed and maintained that a scientist can work in perfect safety, convinced as I was that the date of his publications protected him against any injustice. Unfortunately, I am obliged to admit now that I was utterly mistaken in this regard. I am not dominated by pride and I struggle against this odious vice. Indeed, on publishing my discovery I never for one moment thought of publicity, which could have affected my modesty that I consider one of the first qualities of a scientist. But I certainly cannot accept another, more odious defect, that of the theft of someone else’s scientific property.

The Romanian scientist and other researchers denounced the infringement of Paulescu’s intellectual property rights in several occasions:

A.1. In a letter Paulescu sent to the president of the Nobel Institute on November 6, 1923 (Fig. Suppl. S6) [56].

We transcribe some excerpts of the letter, translated from French:

(...) I beg to be allowed to protest against the fact that this distinction [the Nobel] has been awarded to people who do not deserve it. Indeed, the discovery of these physiological and therapeutic effects belongs entirely to me. (...) These articles do nothing but repeat what I had already said, well-in advance, about the decrease in hyperglycemia and glycosuria, blood and urinary urea, acetonemia and acetonuria, under the influence of injections of intravenous pancreatic extract in diabetic animals.

In volume IV of the treatise on Medicine by E Lancereaux and NC Paulescu, the Romanian scientist wrote:

Before, I believed that the scientist works safely because of the conviction that the date of publication protects him from any injustice. Unfortunately, today I am forced to admit that I was completely wrong [57].

On October 30, 1969, SM Milcu (Vicepresident of the Romanian Academy) and Ion Pavel (President of the Section of Diabetes and Nutritional Diseases, Romanian Academy) wrote to Arne Tiselius, Director of the Nobel Institute, asking for recognition of NC Paulescu as posthumous discoverer of the anti-diabetic hormone [58]. They also mentioned that in the first paper published by Banting and Best in February 1922 [59], the Canadian researchers incorrectly reported Paulescu’s earlier work [49]. We reproduce some excerpts of the letter:

(…) Paulescu’s comprehensive paper, which is a model of scientific research (…) is dated August 31, 1923, which definitely proves that he is the discoverer of insulin. Actually, Banting and Best added nothing in their article published 8 months later (…). We found a basic error in Banting’s work which could certainly have influenced the decision for the 1923 Nobel Prize. On page 253 of his article of February 1922, the author, who was acquainted with Paulescu’s work and cited his work, misrepresented the sense of the results concerning the effect of the aqueous pancreas extract obtained by the Romanian scientist. Here is their text.

“He (Paulescu) stated that injections into peripheral veins produced *no* effect and his experiments show that second injections do not produce such marked effects as the first*.*” Paulescu, however, wrote exactly the contrary as may be seen from the conclusions on his paper comprising experiment IV (August 1921).

Paulescu’s original text read [49]: “Les mêmes effets, c’est-à-dire une diminution ou même une suppression passagère de l’hyperglycémie et de la glycosurie, s’observent aussi lorsqu’on injecte l’extrait pancréatique, non plus dans une veine périphérique, mais dans une branche de la veine porte, par exemple: dans une veinule mésaraïque ou dans une veinule splénique”. Uncertainty persists as to whether Banting and Best's misrepresentation of Paulescu’s results was deliberate or an error due to the Canadian researchers' poor knowledge of French. The historian Michael Bliss wrote the following about this unfortunate incident: “Could it have been a deliberate denigration of a competitor, potentially a scientific scandal? Well, in my hand was the index card containing Charles Best’s 1921 precis of the key Paulescu article, which had been published in French. There on the card was Best’obvious translation error, mistaking the words *non plus as meaning no good*” [60: pp. 203–204].

The Director of the Nobel Institute answered Milcu and Pavel’s letter on December, 29, 1969, stating that, according to the statutes of the Nobel Foundation, the Nobel Prize can only be awarded to shortlisted candidates, but Paulescu had not been nominated. Tiselius added that, in his opinion, Paulescu also deserved the prize [61].

A significant number of experts from different countries publicly expressed their support for Paulescu's priority in the discovery of the antidiabetic hormone, agreeing that Paulescu's results published in August 1921 were practically identical to those published by Banting and Best in February 1922. Some examples are mentioned below.

Alfredo Sordelli, from Bernardo Houssay's research group, was Professor of Biological Chemistry at the Buenos Aires School of Medicine, President of the Academy of Physical and Natural Sciences of the Harvey Society, Academy of Sciences (New York) and of the Société de Biologie de Paris. In collaboration with Juan T. Lewis, he declared in 1924 that Paulescu's results, published in August 1921, were practically identical to Banting and Best’s February 1922 publication [62].

Ian Murray, Professor of Physiology at Anderson College of Medicine in Glasgow and co-founder of the IDF found compelling evidence that several researchers had obtained pancreatic extracts able to improve experimental diabetes in depancreatized dogs, in anticipation of the experiences at the University of Toronto. According to the Scottish researcher, among all these pioneering contributions, the most significant was that of Nicolae Paulescu, who Murray thought to be the discoverer of the antidiabetic hormone (pancrein) [63, 64].

Murray stated that the work of Banting and Best was to be described as confirming Paulescu's discoveries: “Pancrein and insulin are identical (…). The recognition of the work of Paulescu, a distinguished Romanian scientist, has been entirely insufficient. As the Toronto team began their research, Paulescu had already successfully produced the antidiabetic pancreatic extract from the pancreas and demonstrated its efficacy in reducing hyperglycemia in diabetic dogs” [65].

During the celebration in Buenos Aires of the seventh congress of the IDF, a work commission on the discovery of insulin was set up. We reproduce the following extract of the report issued by the commission: “Banting and Best did not isolate insulin. What they did was to produce for the first time pancreatic extracts containing that substance which were suitable for subcutaneous injection into animals and men, such treatment being effective in controlling the symptoms of diabetes mellitus in diabetic dogs and human patients (…). There can be little doubt that Paulescu, as well as Banting and Best, obtained a pancreatic extract which contained insulin, and that the pancrein and the insulin present in the crude extracts in which the hormone was first obtained, are the same substance” [66].

Eric Martin, Professor of Medicine at the University of Geneva, stated in 1971: “There is not the slightest doubt that Paulescu was the first to demonstrate, in an exemplary way, the antidiabetogenic and antiketogenic effect of the pancreatic extract. (…) We must insist on the cardinal importance of Paulescu's discovery, a discovery that Canadian doctors were aware of, but that they interpreted incorrectly, with which certain studies by the Romanian physiologist remained hidden in the shadows” [67].

## The purification of the antidiabetic hormone could not be completed prior to the development of insulin

The patented pancreatic extracts with antidiabetic effects that preceded insulin (acomatol, pancreina) did not spread to general medical practice due to the potential risks of the observed side effects (fever, sweating, vomiting, muscle hypertonia, stomatitis, local “aseptic abscess” and hypoglycemia), attributable to polluting substances, mainly of a protein nature [27, 28, 68].

In the case of acomatol, the explosion of the First World War interrupted a very promising phase of the project, in which, mainly thanks to the contribution of chemical engineer Camille Reuter, the extract obtained had greater biological potency and minimal toxicity. Furthermore, Zülzer’s recruitment into the war, Hoffmann La Roche’s cancellation of the funding and, years later, Zülzer’s expulsion from the university and his professional disqualification by the National Socialist authorities, dictated the end of his research activities.

Zülzer’s expulsion from the University of Berlin and from Lankwitz hospital was the consequence of racial repression by the National Socialist authorities, determined by the “Enabling Act of 1933” (“Ermächtigungsgesetz vom 24. März 1933”, officially titled “Gesetz zur Behebung der Not von Volk und Reich”) and the “Law for the Restoration of the Civil Service” (“Gesetz zur Wiederherstellung des Berufsbeamtentums”), which excluded Jews and other political opponents of Nazism from all civil service positions in Germany. Zülzer had adopted the Protestant religion after his father’s death, but the German Minister of Culture revoked Zülzer’s teaching license on November 24. The vast majority of doctors at Lankwitz Hospital (almost all Jews) were fired. The list of members of the Charité, Medical School of the University of Berlin, who were expelled between 1933 and 1935 included more than 160 teachers and researchers and more than 30 health workers; some were killed by the Nazis or committed suicide [69].

In the case of pancreina, NC Paulescu, working alone with precarious means and failing health, could not make any meaningful progress in the purification of the extract. Paulescu’s radical antisemitism and extreme Orthodox Christianity facilitated his fall into oblivion with the rise to power of the Communist Party. Paulescu, cofounder of right-wing political organizations, was erased from the history of Romanian science for a long time [18, 70].

## Synoptic review of the main contributions toward the development of insulin in the Departments of Physiology and Medicine at the University of Toronto (1921–1923)

### John James Rickard Macleod

John James Rickard Macleod (1876–1935), Professor and Chair of the Department of Physiology at the University of Toronto, delivered a keynote address to the Association of American Physicians in Washington DC on May 3, 1922. In this lecture, Macleod described the main experiments carried out under his coordination between May 1921 and April 1922 and published by the following researchers who agreed to be listed in alphabetical order: FG Banting, CH Best, WR Campbell, JB Collip, AA Fletcher, JJR Macleod, and EC Noble.

1. Effects of insulin in hyperglycemic situations induced by various experimental procedures: Claude Bernard’s *piqûre diabétique*, subcutaneous injection of adrenaline, mechanical asphyxia and carbon dioxide poisoning [71–75].
2. Glycogen and fat deposits in the liver and other organs of diabetic animals and effects of insulin administration on those deposits [74].
3. Effect of insulin on the respiratory quotient in pancreatectomized dogs and in patients with diabetes [76].
4. In the summer of 1922, JJR Macleod carried out a total of 18 experiments in which he observed the serial changes in blood glucose at programmed intervals (Shaffer-Hartmann method) induced in healthy rabbits by subcutaneous injection of:
  pancreatic extracts from fish with a cartilaginous skeleton or elasmobranchs (diffuse distribution of the islets in a compact gland close to the duodenum),
  pancreatic islets of bony or teleost fish (islet tissue separated from the acinar in clearly recognizable nodules),
  pancreatic acinar tissue, free of islets, of teleost fish.
  These experiments were the first showing direct evidence that the production of the antidiabetic hormone is selective of the islet of Langerhans [77].

### James Bertrand Collip

James B. Collip (1892–1965) eliminated lipid impurities from the pancreatic extract by double extraction with sulfuric ether and protein impurities by precipitation with 80% ethyl alcohol, followed by centrifugation. Finally, Collip achieved the isolation of the active principle by selective precipitation with 95% ethanol (January 19, 1922).

On September and October, 1923, respectively, American patents were granted to the semipurified pancreatic extract of Collip, crediting as inventors FG Banting, CH Best and JB Collip, and as owners the Governors of the University of Toronto [78, 79].

### Effective Introduction of Antidiabetic Hormone in the clinic (1922)

The success achieved by the administration of Collip's pancreatic extract to Leonard Thompson and six additional patients, admitted to Toronto General Hospital (TGH) in February 1922, was published in March of the same year [80].

Before the end of 1922, more than fifty diabetic patients had successfully received insulin treatment at the Diabetes Clinic of the TGH by Walter R. Campbell, Andrew A. Fletcher and Frederick G. Banting, under the supervision of Prof. Duncan A. Graham [81, 82].

## The priority in scientific  discoveries: analysis and controversies

For a researcher, communicating a discovery that contributes to the advancement of scientific knowledge is a matter of the utmost importance, and the recognition of the priority of the discovery by institutions and the academic community represents a main achievement. It is therefore not surprising that the determining principles of the attribution of priority have been from time immemorial, and continue to be so today, a matter of controversy.

### Robert King Merton (1910–2003): Organization of science and priority rule

Robert K. Merton (original name, Meyer Robert Schkolnick), was born in Philadelphia, into a humble Jewish family, who immigrated to the United States from Eastern Europe. At Harvard, George Sarton, Professor of Sociology, directed his doctoral thesis, titled *Science, Technology and Society in Seventeenth Century England* [83].

Merton spent the rest of his academic life as a professor at Columbia University (New York). His text *Social Theory and Social Structure* is a landmark publication in sociology, translated to over 20 languages. In 1998, the International Sociological Association listed this book as the third most important sociological book of the twentieth century [84].

Merton explained the origin of modern science, identified the set of values and norms *(ethos)* that should guide scientists and the reward mechanisms of science. He investigated the originality of creation, plagiarism, tradition and progress in the scientific world and the importance of the contributions of multiple researchers over time toward a certain exceptional discovery, endorsing Newton's famous phrase “On the shoulders of giants” (“If I have come to see further, it was by standing on the shoulders of giants”) [85].

**Fig. 7:**
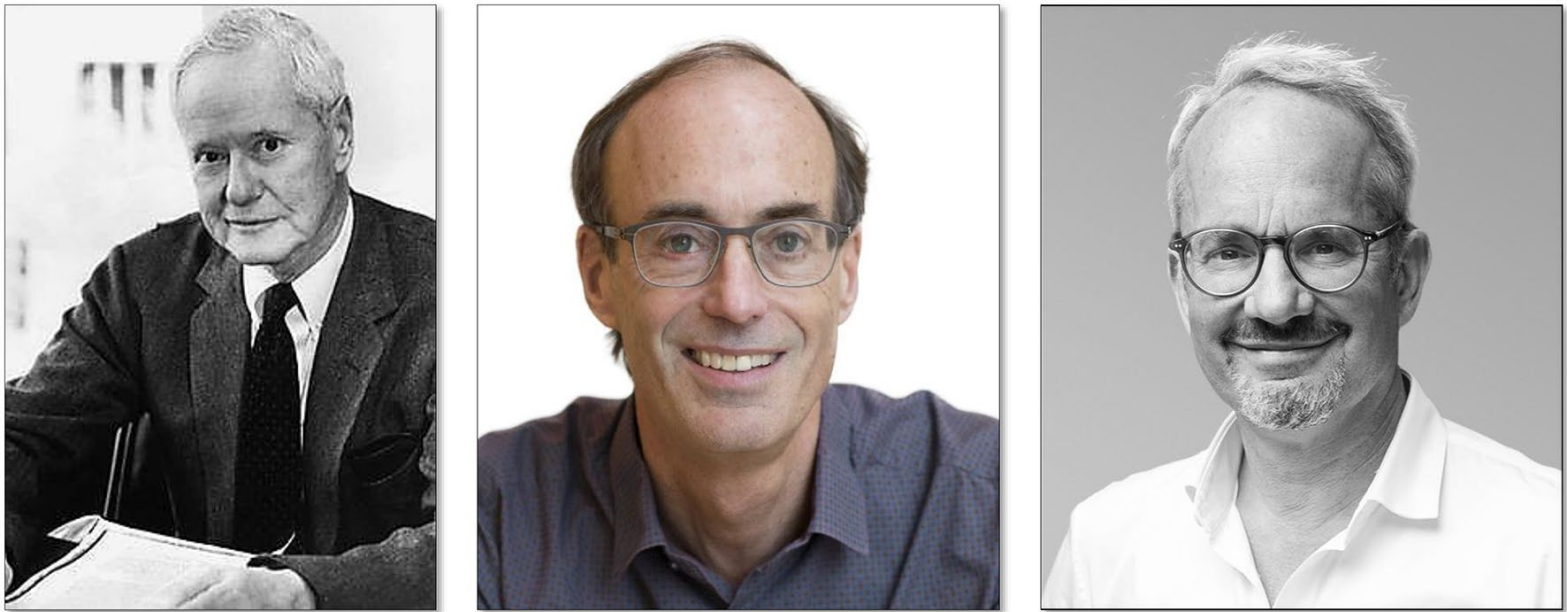
*From left to right*: Portrait of RK Merton. Unknown date and author. Robert K. Merton described in the presidential address of the American Sociological Society (1957) the characteristic values, norms and organization of the institution of science, as well as the basis for the sociological investigation of priority in science. Portraits of Ronald D. Vale, Emeritus Professor of Cellular and Molecular Pharmacology at the University of California, San Francisco, and Vicepresident and Executive Director of the Howard Hughes Medical Institute; and Anthony A. Hyman, director of the Max Planck Institute of Molecular Cell Biology and Genetics

In his presidential address at the annual meeting of the American Sociological Society (1957) Merton defined the **priority rule** as the credit granted to an individual or a group of individuals who made the discovery in the first place, documenting it through an original publication. The priority date corresponds to the date on which the publication was sent. Certified knowledge is legitimized by the institution of science through codification norms and disclosure of discovery (letters, books, articles in scientific journals) (Fig. 7) [86].

According to R.D. Vale (UC San Francisco) and Anthony Hyman (Max Planck Institute) (Fig. 7), the recognition of the primacy of a scientific discovery requires satisfactory fulfillment of the accreditation requirements of two decisive factors: revelation of the discovery and validation by the scientific community [87, 88].

The recognition of priority of the scientific discovery must avoid behavioral deviations regarding its originality *(fraud, plagiarism, fabrication of non-existing data and exclusion of unsatisfactory results)* [89].

Examples of behavioral deviation by FG Banting and CH Best are described below:

A—On February 5, 1923, Paulescu wrote to FG Banting, asking for cooperation and enclosed with the letter his 1921 publications showing the positive results of pancreina on experimental diabetes. Banting never answered. On November 6, 1923, Paulescu bitterly protested against the concesion of the Nobel Award to Banting and Macleod and stated that the Toronto team had infringed his intellectual property rights [29].

B—Banting and Best’s first article contained formal errors, meaningful mistakes and discrepancies between text and figures. *The main figures of the manuscript showed that the administration of the “degenerated pancreas extract” only achieved a partial decrease in the levels of blood and urine glucose in only some of the depancreatized dogs*. Nevertheless, Banting and Best felt that these results justified stating that the extract contained the internal pancreatic secretion. JJR Macleod decided not to sign the manuscript as coauthor [29: pp. 175–176]. The transcription of the satisfactory results only and *the exclusion of the unsatisfactory results is a manifestation of dishonesty and constitutes an error* [90]. Besides, as mentioned a few pages above, Banting and Best incorrectly reported Paulescu’s results.

The physiologist Ffrangcon Roberts (University of Cambridge) published a letter to the editor in the *British Medical Journal* criticizing Banting and Best’s experiments. He qualified them as “badly designed, badly conducted and badly interpreted”. Michael Bliss corroborated and expanded on that comment: “Banting and Best’s research was so badly done that, without the help of Macleod and Collip, and a much more subtle view of the constituents of the discovery of insulin, the two young Canadians would be fated to disappear from medical history” [91].

### The award of the Nobel Prize in Physiology or Medicine, 1923

The award of the Nobel Prize in Physiology or Medicine in 1923 to Banting and Macleod was peculiar in several respects.

1. Banting was the first Canadian to receive the award.
2. The awarding of the Nobel to a candidate in the first year in which it was proposed had only happened previously to four laureates: in 1901, Nobel awarded to Emil A. von Behring for his work on serum therapy, especially for its application to diphtheria; in 1912 to Alexis Carrel for his contributions to vascular suturing and to transplantation of blood vessels and organs; and in 1922, Nobel awarded jointly to Archibald V. Hill for his discoveries in muscle thermodynamics and to Otto F. Meyerhof for his discovery on the relationship between oxygen consumption and lactic acid metabolism in muscle [92].
3. The limited information available to the members of the Nobel Committee on the nominations received, consisting of a small number of documents.
4. The researchers who protested the decision of the Nobel Committee (G.L. Zülzer, E.L. Scott, J. Murlin, N.C. Paulescu, C.H.Best) were not on the list of nominees. According to the statutes, that’s the reason why they were not eligible (although there have been cases where Nobel awards were granted in the absence of external nominations, in which case, the candidates were proposed by members of the Nobel Committee themselves). Interestingly, one of them was the 1920 Nobel, awarded to August Krogh, who was nominated by the chairman of the Nobel Committee and professor at the Karolinska Institute, Johan E. Johansson.
5. The postulate of the "greatest benefit to humanity during the year prior to the date of the award", included in Alfred Nobel's will, was undoubtedly a factor of definite importance. However, the purification process that made the difference with regard to the extracts tested by other authors previously, was an achievement attributable in 1922, almost exclusively, to James Bertrand Collip, absent from the list of nominees. This clause strictly excludes all alternative candidates, with the exception of C.H. Best. However, the reality is that the deliberation of the commission and international experts takes time (an exceptional case was that of Peyton Rous, who discovered the tumor virus in chickens in 1916 and received the award 50 years later). For this reason, the current interpretation for the Nobel Foundation is that "the benefits of the discovery have been apparent to the members of the Karolinska Institute during the previous year" [4].
6. As for the "scientific value" of the discovery, the question is more complex. Insulin does not meet the required criteria ("discover something hidden, unknown or secret"), as it was the last of the organotherapeutic preparations with antihyperglycemic activity investigated between 1889 and 1922.
7. It is essential to differentiate the terms "discovery of the antidiabetic hormone" and "discovery of insulin". The greatest contribution of the researchers from the University of Toronto was *the purification of the extract, making it suitable for clinical use*. This was recognized in 1971 by the Special Committee of the IDF, editor of the report on the investigations related to the discovery of the antidiabetic hormone [66]. In the third edition of the text *Nobel, The Man and his Prizes* (1972) revised by Carl Gustaf Bernhard, professor at the Karolinska Institute between 1948 and 1971 and permanent secretary of the Royal Swedish Academy of Sciences between 1973 and 1981, the Nobel Prize of Physiology or Medicine, 1923, was given “to the credit for having produced the pancreatic hormone in a practical available form” [93: pp. 224–225].

## Who discovered the antidiabetic hormone? Proposals of the authors by way of conclusions

The discovery of the antidiabetic hormone (acomatol, pancrein, insulin) has been the result of a complex process, extended over time, through different stages and contributions from multiple actors. The answer to the priority dispute may differ according to the following considerations [22, 28, 29]:

A. If we consider that the most important achievement was the first demonstration of the beneficial effect of the administration of pancreatic extracts to an animal with experimental diabetes, credit must go to the pioneer research of Eugène Gley (1900).
B. If we apply the criteria formally established by the *Priority Rule* (RK Merton, R Vale and A. Hyman), the primacy should be assigned to GL Zülzer (first publication in 1906 and first patents for acomatol in 1908, 1909 and 1912).
C. If we estimate that primacy corresponds to the first demonstration that the pancreatic extract exerts antidiabetic effects in humans (even with collateral toxicity), credit must go to GL Zülzer (1908).
D. If we place greater importance on the scientific quality of the investigations regarding the physiological basis of the endocrine action of the pancreas, with the highest excellence in the descriptions of the experimental method and the metabolic and beneficial effects exerted by the pancreatic extract on experimental diabetes, the priority of discovery should be shared by NC Paulescu (1916) and JJR Macleod (1922).
E. If the elected alternative is the greatest benefit for humanity, the primacy would correspond to the success in the purification of the extract and its introduction in the clinic by JB Collip, WR Campbell and AA Fletcher (1922).

## Supplementary Information

Below is the link to the electronic supplementary material.

**SupplFig1 Left:** August and Marie Krogh. Author and date unknown. Source: Underwood & Underwood /Det Kgl. Library (Public domain). **Right:** Portrait of Hans Christian Hagedorn. Author and date unknown. Source: https://www.novonordisk.com/ (Public domain) **SupplFig2 Left:** Portrait of Göran Liljestrand. Unknown date and author. Source: Nobel Foundation. **Right:** Extract from the handwritten (in Danish) from Krogh to Liljestrand dated Junuary 20, 1923. (Digital reproduction on the Nobel Foundation website) **SupplFig3** University of Toronto Governing Board invitation to the Banting and Macleod Banquet in honor of the discovery of insulin and the award of the 1923 Nobel Prize in Physiology or Medicine. Pictured from left to right: JG Fitzgerald, Albert Gooderham, Dean Alexander Primrose, WF Nickle, CH Best, Sir William Mulock, FG Banting, Canon HJ Cody, Sir Edmund Walker, JJR Macleod, Sir Robert Falconer (University of Toronto Library Archives) **SupplFig4** Georg L Zülzer. The overcoming of diabetes (1923) **SupplFig5** Georg L Zülzer. “Über Acomatol, das deutsche Insulin” (1923) **SupplFig6** NC Paulescu. Protest letter to the President of the Nobel Institute, against the awarding of the Nobel Prize in Physiology or Medicine, 1923, to FG Banting and JJR Macleod **SupplFig7** Milcu and Pavel. Letter to the Director of the Nobel Institute **SupplFig8** A. Tiselius’ Letter to SM Milcu and Ion Pavel (PPTX 20501 kb)
