# Supplementary figures and images for: The Nobel Prize of Physiology or Medicine, 1923: controversies on the discovery of the antidiabetic hormone
Source: Acta Diabetol. 2023 Jun 2;60(9):1241–56. doi: 10.1007/s00592-023-02098-9 (PMC10359383; doi:10.1007/s00592-023-02098-9)

## Slide 1
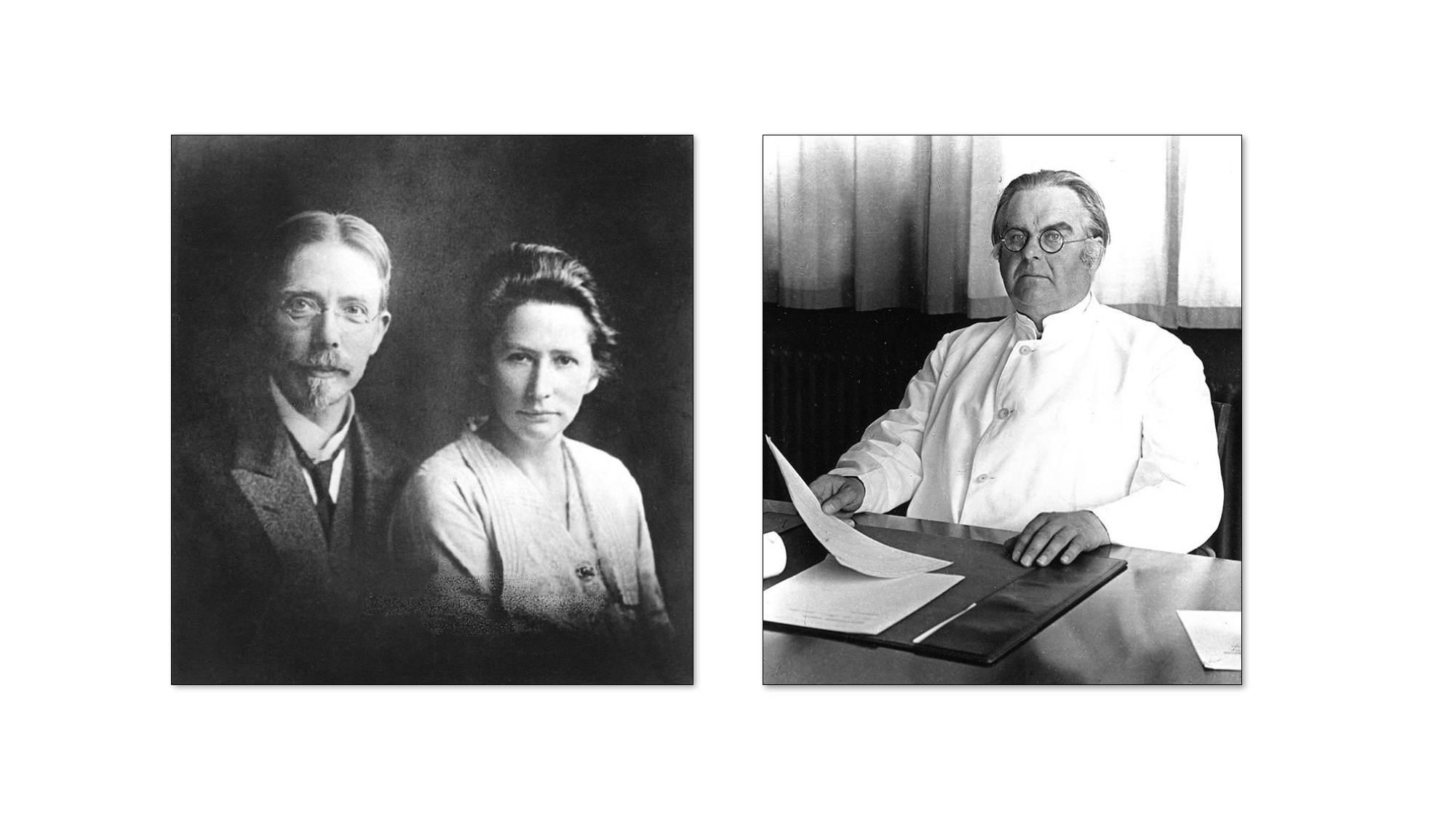

## Slide 2
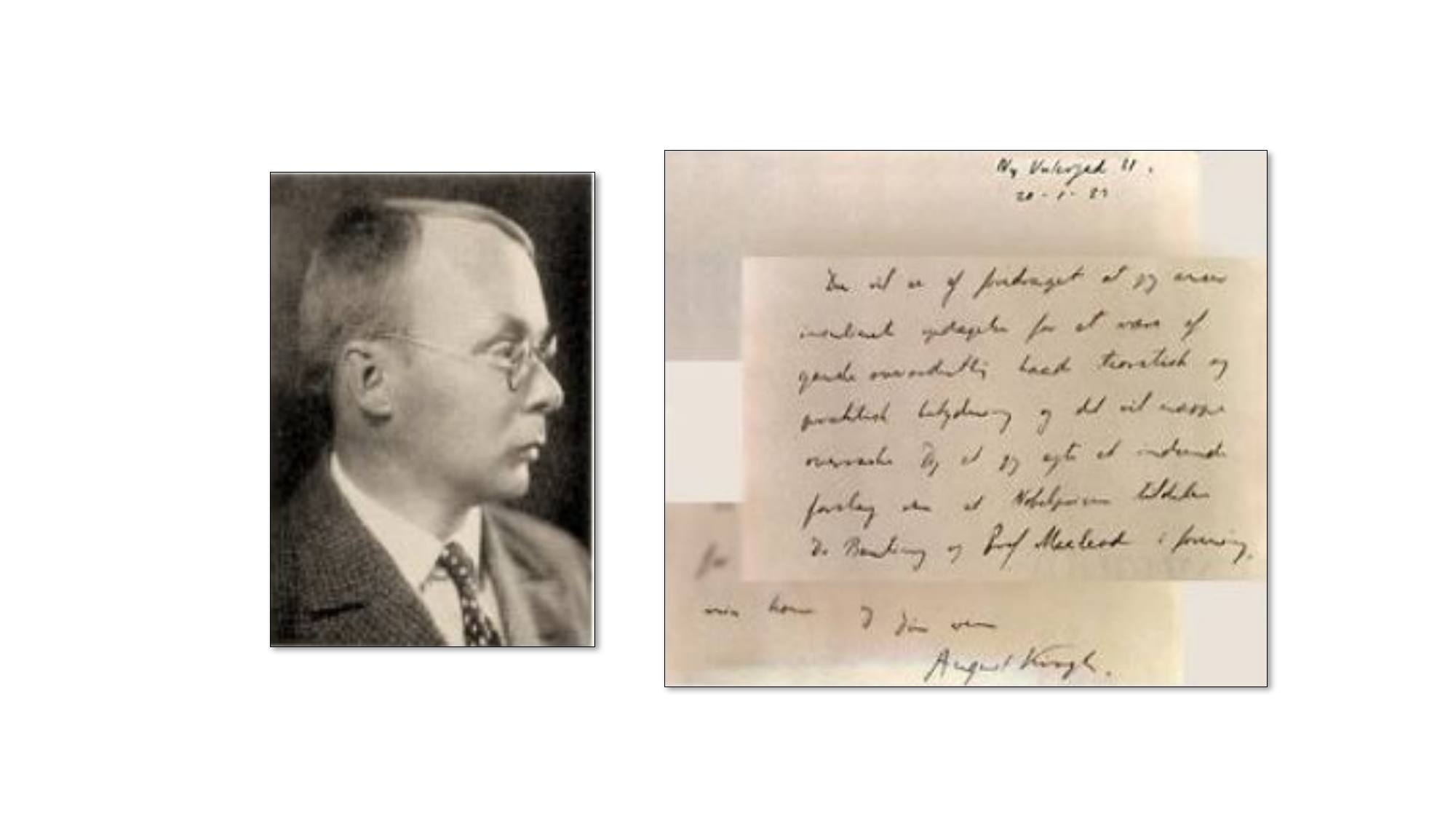

## Slide 3
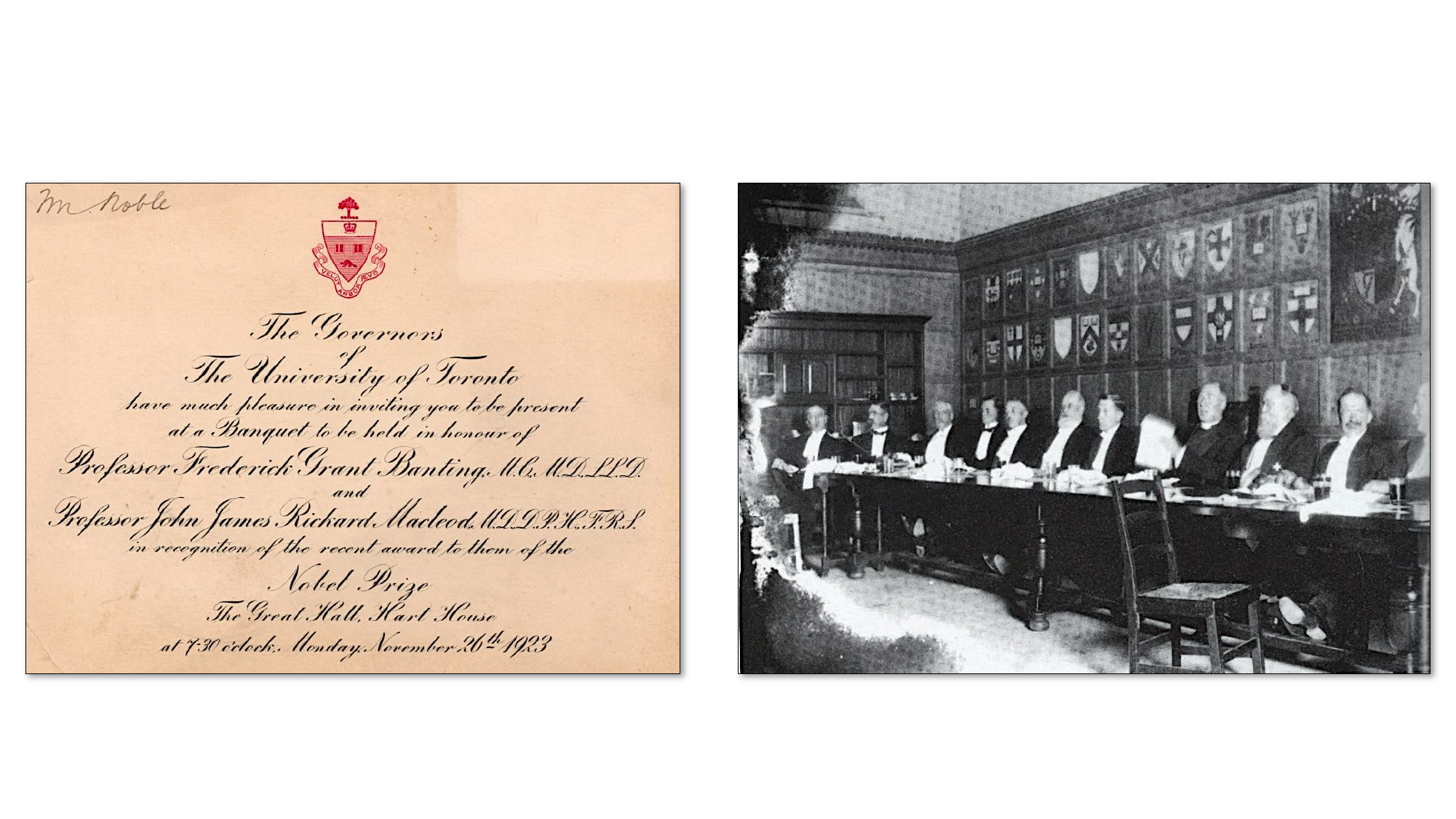

## Slide 4
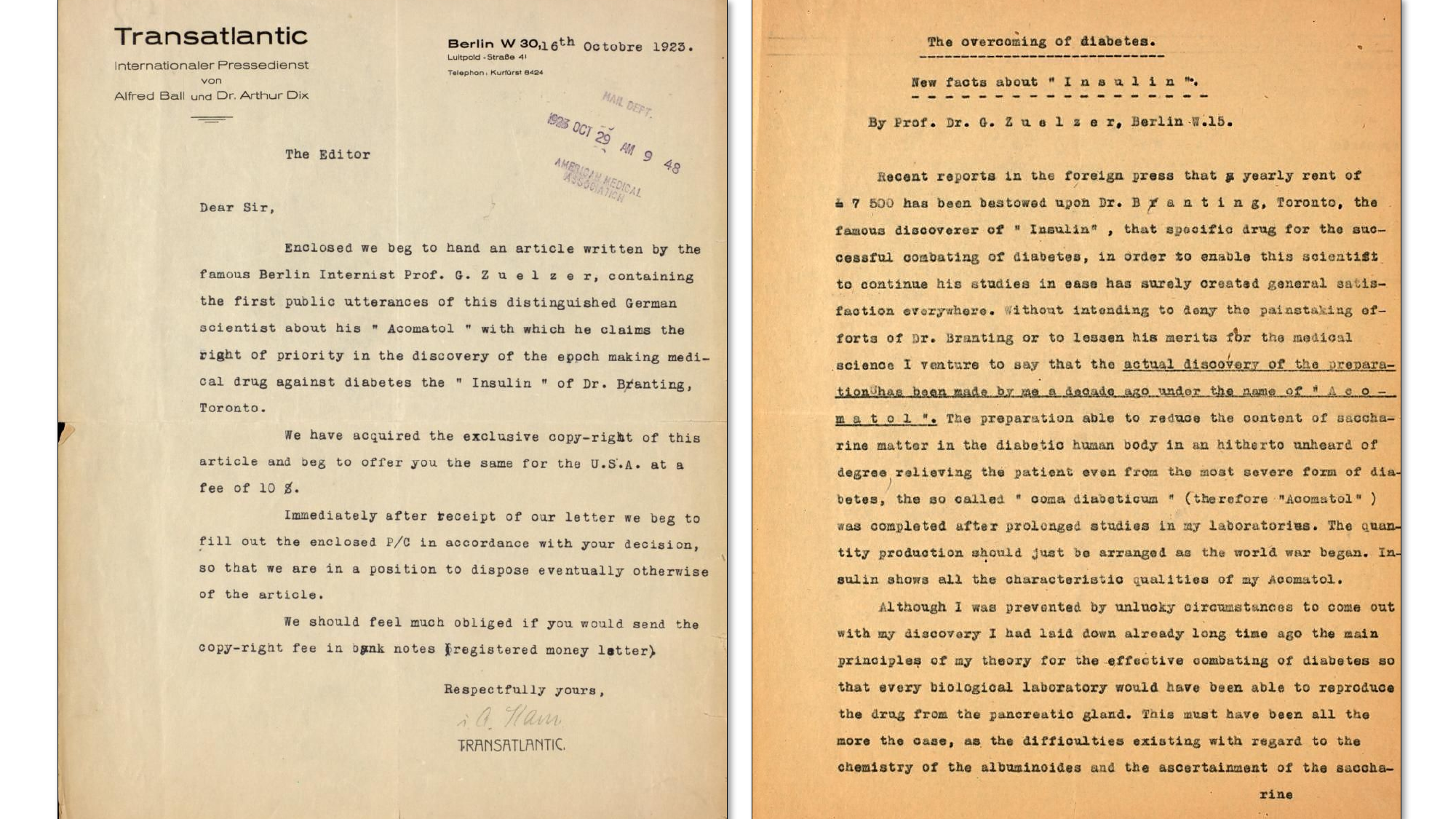

## Slide 5
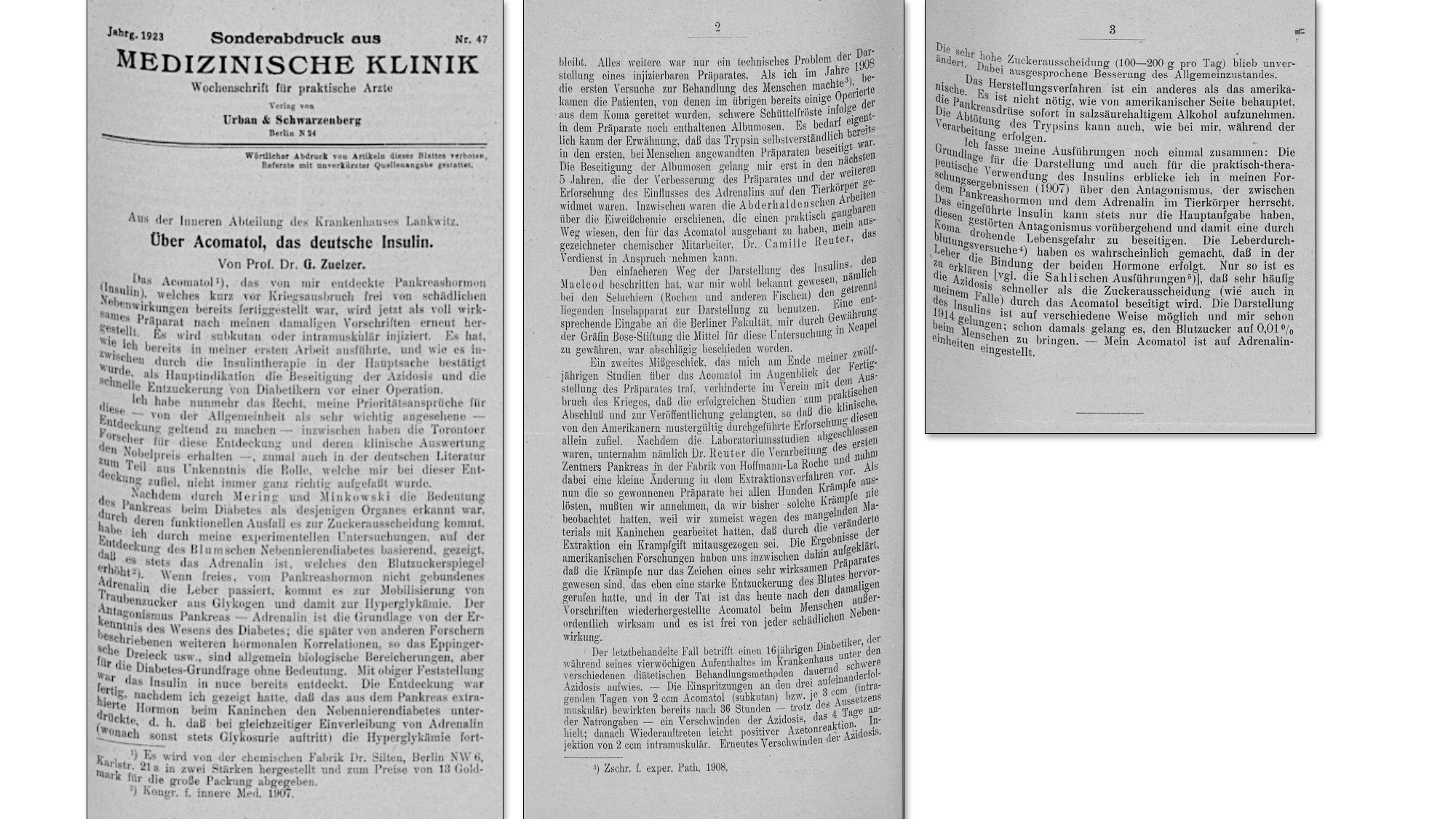

## Slide 6
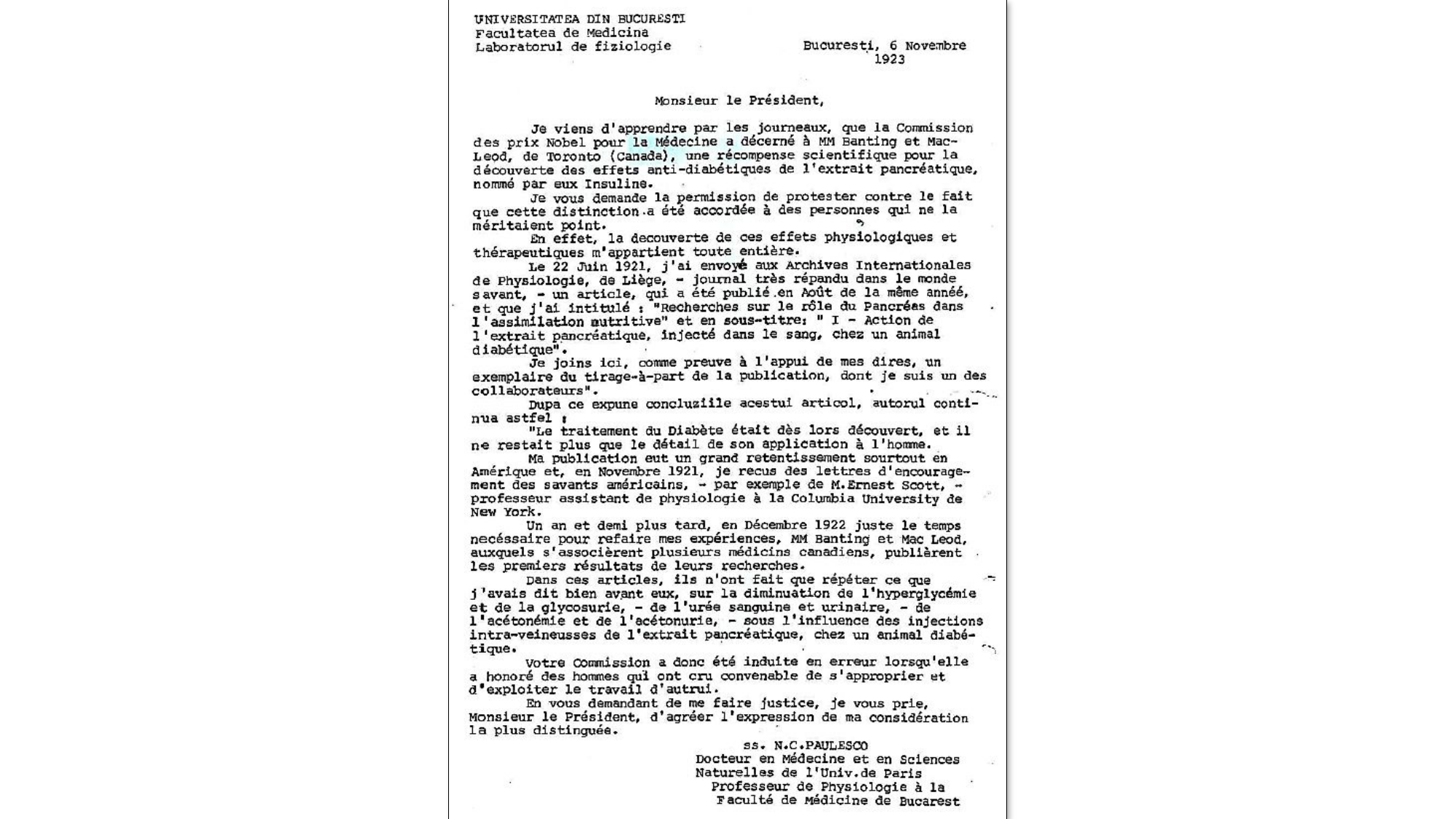

## Slide 7
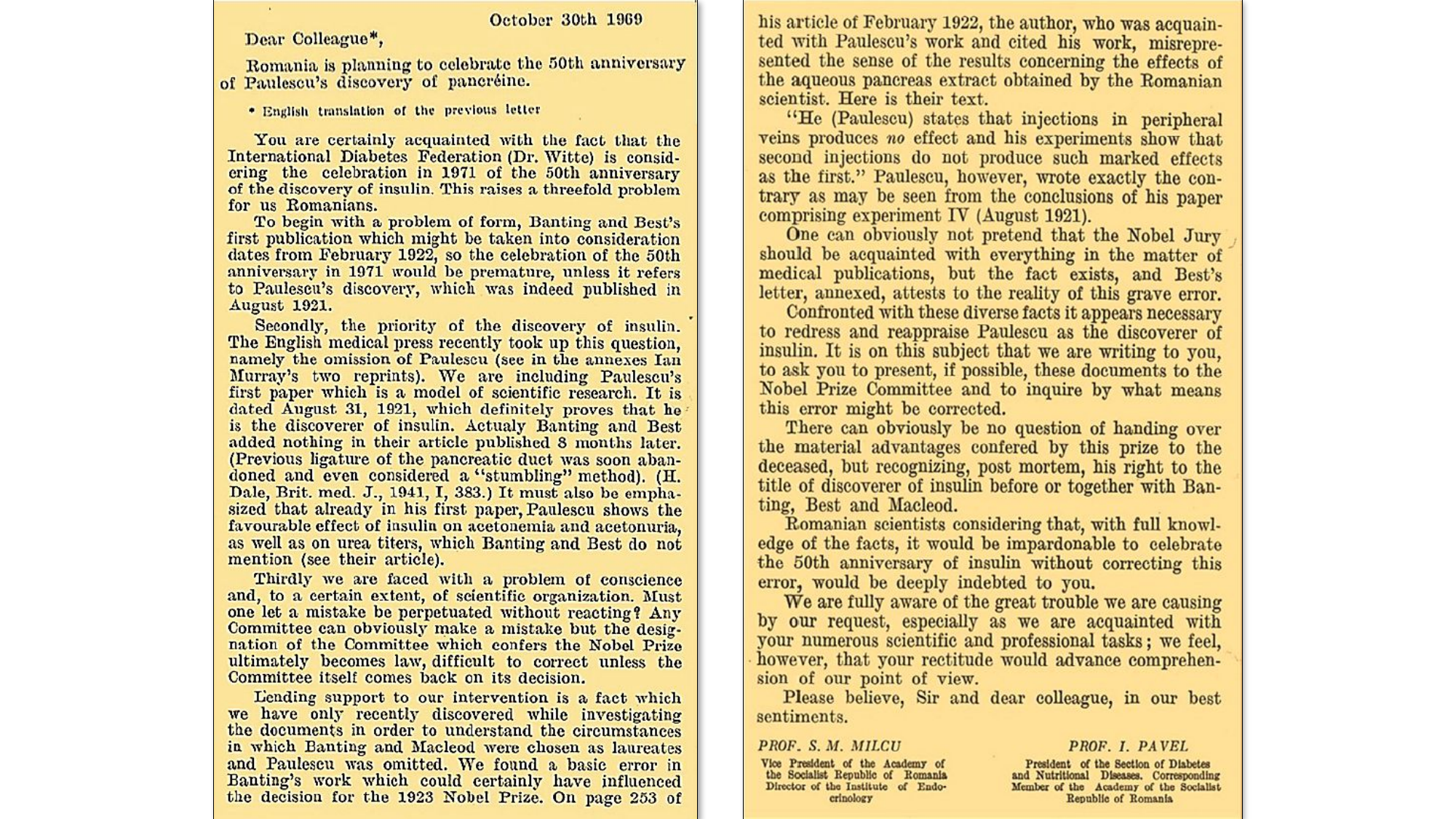

## Slide 8
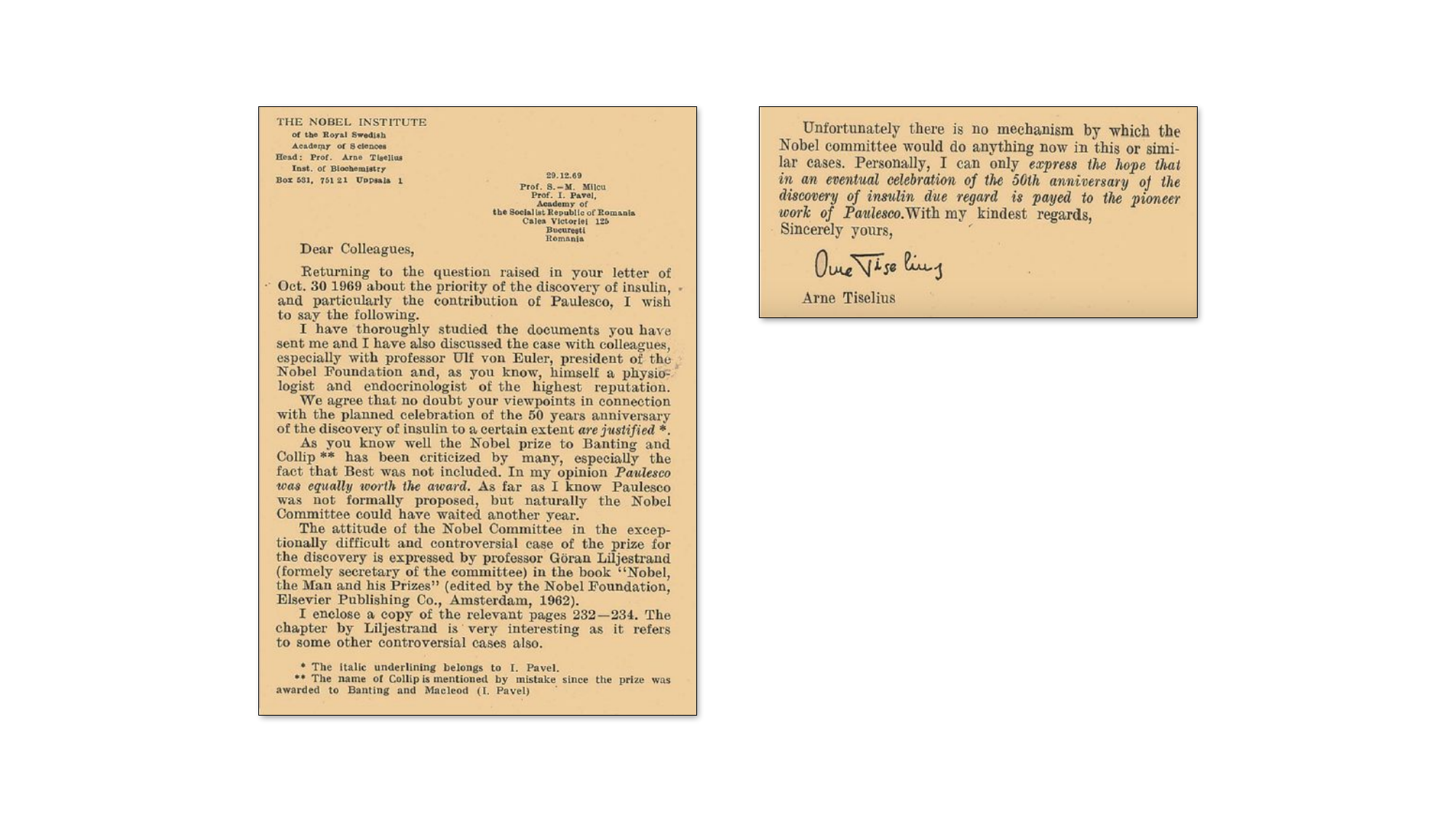

Supplement: Supplementary file 1 — SupplFig1 Left: August and Marie Krogh. Author and date unknown. Source: Underwood & Underwood /Det Kgl. Library (Public domain). Right: Portrait of Hans Christian Hagedorn. Author and date unknown. Source: https://www.novonordisk.com/ (Public domain) SupplFig2 Left: Portrait of Göran Liljestrand. Unknown date and author. Source: Nobel Foundation. Right: Extract from the handwritten (in Danish) from Krogh to Liljestrand dated Junuary 20, 1923. (Digital reproduction on the Nobel Foundation website) SupplFig3 University of Toronto Governing Board invitation to the Banting and Macleod Banquet in honor of the discovery of insulin and the award of the 1923 Nobel Prize in Physiology or Medicine. Pictured from left to right: JG Fitzgerald, Albert Gooderham, Dean Alexander Primrose, WF Nickle, CH Best, Sir William Mulock, FG Banting, Canon HJ Cody, Sir Edmund Walker, JJR Macleod, Sir Robert Falconer (University of Toronto Library Archives) SupplFig4 Georg L Zülzer. The overcoming of diabetes (1923) SupplFig5 Georg L Zülzer. “Über Acomatol, das deutsche Insulin” (1923) SupplFig6 NC Paulescu. Protest letter to the President of the Nobel Institute, against the awarding of the Nobel Prize in Physiology or Medicine, 1923, to FG Banting and JJR Macleod SupplFig7 Milcu and Pavel. Letter to the Director of the Nobel Institute SupplFig8 A. Tiselius’ Letter to SM Milcu and Ion Pavel (PPTX 20501 kb) [file 592_2023_2098_MOESM1_ESM.pptx]
